# Reply to Allaire, M.; Thabut, D. Comment on “Wu et al. Baveno VII Criteria Is an Accurate Risk Stratification Tool to Predict High-Risk Varices Requiring Intervention and Hepatic Events in Patients with Advanced Hepatocellular Carcinoma. *Cancers* 2023, *15*, 2480”

**DOI:** 10.3390/cancers16040725

**Published:** 2024-02-09

**Authors:** Claudia Wing-Kwan Wu, Terry Cheuk-Fung Yip, Vincent Wai-Sun Wong, Grace Lai-Hung Wong, Ken Liu, Rashid Nok-Shun Lui

**Affiliations:** 1 Medical Data Analytics Centre, Hong Kong SAR, Chinawongv@cuhk.edu.hk (V.W.-S.W.);; 2 Department of Medicine and Therapeutics, Prince of Wales Hospital, Hong Kong SAR, China; 3 Institute of Digestive Disease, Prince of Wales Hospital, Hong Kong SAR, China; 4 AW Morrow Gastroenterology and Liver Centre, Royal Prince Alfred Hospital, Camperdown, NSW 2050, Australia

We thank Allaire et al. [1] for their interest and valuable comments regarding our recently published post hoc analysis study using Baveno’s criteria for excluding varices needing treatment (VNT) in the hepatocellular carcinoma (HCC) population. The authors discussed and challenged a few methodological points and our study results, which we will address below.

The authors correctly pointed out that the Baveno VI criteria (LSM < 20 kPa and platelet > 150 × 10^9^/L) is intended to rule out VNT in patients with compensated cirrhosis. The included patients with Child–Pugh B in our cohort did not have a history of decompensating events (e.g., ascites, HE, etc.) but were classified as Child–Pugh B purely based on serological markers, such as low albumin or elevated bilirubin level. Therefore, we found it suitable to include these patients in this study. We have also performed a sub-analysis on Child–Pugh A cirrhosis patients below.

Indeed, the presence of portal (not just vascular) invasion may impact the accuracy of Baveno criteria in predicting high-risk varices. A subgroup analysis on the portal vein thrombosis (PVT) population has already been performed in our original paper. Within our cohort, 12 out of 75 patients (16%) with PVT had VNT, and 33 out of 125 patients (26%) without PVT had VNT. Our study showed that the presence of PVT did not significantly impact the rate of clinically significant varices (*p*-value 0.088). Unfortunately, we do not have the information on the size and number of tumours to perform a subgroup analysis in that aspect.

Allaire et al. pointed out that the high proportion of patients with hepatitis B in our cohort may not represent the global HCC population. In the local Hong Kong population, there is a high prevalence of hepatitis B, with chronic hepatitis B accounting for 80% of all causes of HCC between 1992 and 2016 [2]. This makes our data particularly relevant in Hong Kong and the Asian population. From an epidemiological point of view, the “global HCC population” is represented mostly by Asian and African patients with hepatitis B-related HCC. Most clinical trials with unresectable HCC also had a large proportion of patients with chronic hepatitis B as disease aetiology. However, we understand that our conclusions would need further validation in HCC from other aetiology. As mentioned in our study, HCC from viral hepatitis may perform better than HCC from other aetiology, given that anti-viral therapy can significantly slow liver disease progression and reduce the risk of clinically significant portal hypertension or variceal bleeding risk. MASLD-related HCC population is under-represented in our study and would require further evaluation, especially given its unique mechanism of portal hypertension, which can occur even in early fibrosis or when cirrhosis is absent.

We would also like to clarify that HCC progression was not included as a liver-related event in this study. Hepatic decompensating events were defined as the presence of any one of the following: variceal bleeding, ascites, spontaneous bacterial peritonitis (SBP), hepatic encephalopathy, hepatorenal syndrome (HRS), and liver-related death.

We modified the calculation per the authors’ recommendations regarding the methodological discrepancy in calculating the missed VNT rate. There were also some missing liver stiffness measurement (LSM) data, which have now been recovered; the available data have been incorporated into the analysis, and the most updated results are below.

Within our total sample size of 200 patients, 46 fulfilled the Baveno VI criteria. In total, 40 out of 46 (87%) patients were correctly identified as not having VNT, and 6 out of 46 (13%) had significant VNT missed by Baveno VI criteria. Among the 154 patients who did not fulfil the Baveno VI criteria, 39 out of 154 (25%) had VNT. The sensitivity, specificity, PPV, and NPV of Baveno VI criteria for predicting VNT were 86%, 25%, 25%, and 86%, respectively (Table 1).

A subanalysis of Child–Pugh A patients was performed. Of the 135 patients with Child–Pugh A cirrhosis, 39 fulfilled the Baveno VI criteria. In total, 5 out of 39 (12.8%) patients had VNT missed by Baveno VI criteria. The sensitivity, specificity, PPV, and NPV of Baveno VI criteria for predicting VNT in the Child–Pugh A subgroup were 80%, 31%, 21% and 87%, respectively (Table 2).

A subgroup analysis via BCLC staging was also performed. In patients with favourable Baveno VI criteria, 0 out of 4 (0%) BCLC-A patients, 3 out of 14 (21%) BCLC-B patients, and 3 out of 28 (11%) BCLC-C patients presented with VNT. For patients with unfavourable Baveno VI, 8 out of 21 (38%) BCLC-A patients, 12 out of 37 (32%) BCLC-B patients, and 19 out of 96 (20%) BCLC-C patients presented with VNT (Table 3; Figure 1). Therefore, we concur with the conclusion of Allaire et al. [3] that the Baveno criteria are unsuitable for use in patients with advanced HCC. However, it still holds promise as a non-invasive risk stratification tool for VNT in early-stage HCC populations with small lesions [4]. Nevertheless, more prospective studies must be conducted to further validate this.

## Figures and Tables

**Figure 1:**
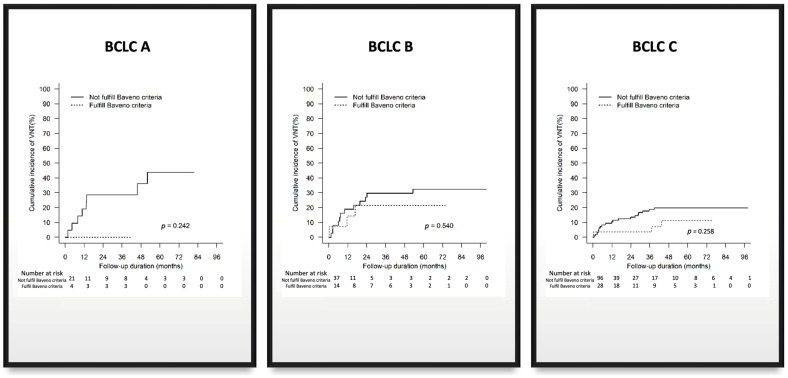
Subanalysis of Baveno criteria by BCLC staging.

**Table 1:** Performance of Baveno VI criteria for the entire cohort (N = 200).

	No VNT	VNT	
Fulfilled Baveno VI criteria	40	6	
Did not fulfil Baveno VI criteria	115	39	
	Value	Lower 95%CI	Upper 95%CI
Sen	0.866667	0.732075	0.949458
Spe	0.258065	0.191206	0.334413
PPV	0.253247	0.186696	0.329544
NPV	0.869565	0.737435	0.950593

**Table 2:** Performance of Baveno VI criteria for Child–Pugh A (N = 135).

	No VNT	VNT	
Fulfilled Baveno VI criteria	34	5	
Did not fulfil Baveno VI criteria	76	20	
	Value	Lower 95%CI	Upper 95%CI
Sen	0.800000	0.592963	0.931689
Spe	0.309091	0.224454	0.404310
PPV	0.208333	0.132145	0.303251
NPV	0.871795	0.725701	0.957032

**Table 3:** Performance of favourable Baveno VI criteria in ruling out VNT, according to BCLC staging.

Criterion	N	Favourable Baveno VI Criteria	Se (%)	Sp (%)	PPV (%)	NPV (%)	VNT Missed
Whole cohort	200	46	86.7% (73.2%–94.9%)	25.8% (19.1%–33.4%)	25.3% (18.7%–33.0%)	87.0% (73.7%–95.1%)	6/46 (13.0%)
BCLC A							
Whole cohort	25	4	100.0% (63.1%–100.0%)	23.5% (6.8%–49.9%)	38.1% (18.1%–61.6%)	100.0% (39.8%–100.0%)	0/4 (0.0%)
Right lobe involvement	11	3	100.0% (15.8%–100.0%)	33.3% (7.5%–70.1%)	25.0% (3.2%–65.1%)	100.0% (29.2%–100.0%)	0/3 (0.0%)
With PVT	1	0	-	-	-	-	-
BCLC B							
Whole cohort	51	14	80.0% (51.9%–95.7%)	30.6% (16.3%–48.1%)	32.4% (18.0%–49.8%)	78.6% (49.2%–95.3%)	3/14 (21.4%)
Right lobe involvement	21	8	75.0% (34.9%–96.8%)	46.2% (19.2%–74.9%)	46.2% (19.2%–74.9%)	75.0% (34.9%–96.8%)	2/8 (25.0%)
With PVT	5	2	50.0% (1.3%–98.7%)	33.3% (0.8%–90.6%)	33.3% (0.8%–90.6%)	50.0% (1.3%–98.7%)	1/2 (50.0%)
BCLC C							
Whole cohort	124	28	86.4% (65.1%–97.1%)	24.5% (16.5%–34.0%)	19.8% (12.4%–29.2%)	89.3% (71.8%–97.7%)	3/28 (10.7%)
Right lobe involvement	59	16	78.6% (49.2%–95.3%)	28.9% (16.4%–44.3%)	25.6% (13.5%–41.2%)	81.2% (54.4%–96.0%)	3/16 (18.8%)

## Data Availability

The data presented in this study are available in this article.
